# Vision and retina evolution: How to develop a retina

**DOI:** 10.1016/j.ibneur.2022.03.008

**Published:** 2022-04-01

**Authors:** Bernd Fritzsch, Paul R. Martin

**Affiliations:** a Department of Biology & Department of Otolaryngology, The University of Iowa, Iowa City, IA, 52242, USA; b Faculty of Medicine and Health, Save Sight Institute & ARC Centre for Integrative Brain Function, University of Sydney, NSW 2006, Australia

**Keywords:** Opsin, Retina, Neuropore, Pineal, Retinal ganglion cell, Eye

## Abstract

Early in vertebrate evolution, a single homeobox (Hox) cluster in basal chordates was quadrupled to generate the Hox gene clusters present in extant vertebrates. Here we ask how this expanded gene pool may have influenced the evolution of the visual system. We suggest that a single neurosensory cell type split into ciliated sensory cells (photoreceptors, which transduce light) and retinal ganglion cells (RGC, which project to the brain). In vertebrates, development of photoreceptors is regulated by the basic helix-loop-helix (bHLH) transcription factor *Neurod1* whereas RGC development depends on *Atoh7* and related bHLH genes. Lancelet (a basal chordate) does not express *Neurod* or *Atoh7* and possesses a few neurosensory cells with cilia that reach out of the opening of the neural tube. Sea-squirts (Ascidians) do not express *Neurod* and express a different bHLH gene, *Atoh8,* that is likely expressed in the anterior vesicle. Recent data indicate the neurosensory cells in lancelets may correspond to three distinct eye fields in ascidians, which in turn may be the basis of the vertebrate retina, pineal and parapineal. In this review we contrast the genetic control of visual structure development in these chordates with that of basal vertebrates such as lampreys and hagfish, and jawed vertebrates. We propose an evolutionary sequence linking whole-genome duplications, initially to a split between photoreceptor and projection neurons (RGC) and subsequently between pineal and lateral eye structures.

## 1 How to make an eye from scratch

Opsins are central to phototransduction as well as to understanding evolution of the visual system (Arendt et al., 2016, Nilsson, 2021, Schwab, 2018). Opsins evolved together with the regulatory gene *Pax6* that interacts with several other genes during eye development (Fritzsch and Piatigorsky, 2005, Schlosser, 2021, Schwab, 2018). A second and largely independent control mechanism comprises retinoic acids (RA) and their synthesizing *Raldh* enzymes (Crandall et al., 2011, Neitz et al., 2021). A third and equally important aspect of vertebrate eye development is the process of lateral evagination from the neural tube; each evagination interacts with a lens placode to develop a retina and central projections from retinal ganglion cells (Fuhrmann, 2010, Lamb, 2013, Schwab, 2018). In addition to retinal ganglion cells, the developing vertebrate retina needs to generate, in sequence, horizontal, cone, amacrine, rod, bipolar and Müller cells (Lamb, 2013). Finally, additional molecular, development and neuronal control mechanisms work to transform some retinal ganglion cells to yield a melanopsin-based phototransduction cell family with unique properties and brain connections (Lucas et al., 2020).

**Fig. 1:**
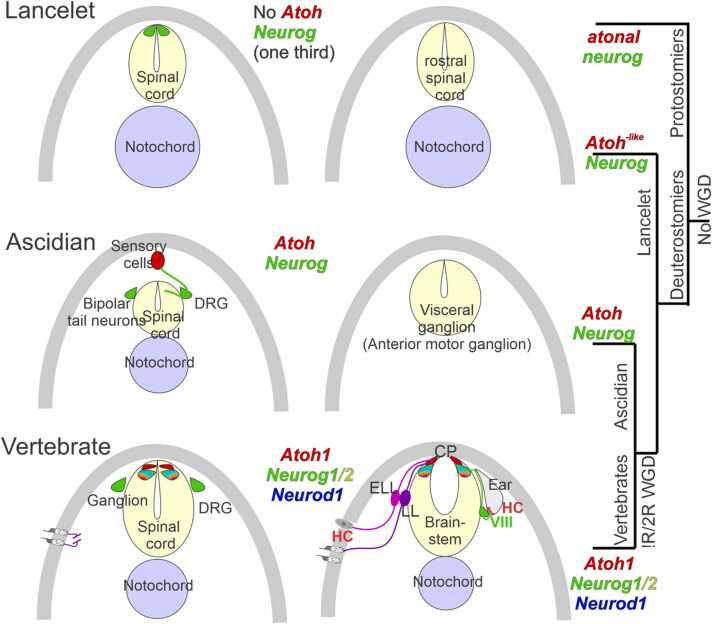
Comparing lancelet, ascidians, and lamprey in the spinal cord/brainstem. Lancelets show a limited expression of the bHLH Neurog and have no *Atoh*-like gene (top). Ciona has sensory cells that require *Atoh* and are innervated by bipolar tail neurons expressing *Neurog* which extend to reach the visceral ganglion (middle, DRG). Vertebrates have dorsal root ganglia that depend on *Neurog1*/2, which is also expressed in *Atoh1*, *Neurog1*/2 and *Neurod1* in the spinal cord and the brainstem (bottom). The brainstem is innervated by electroreceptor (ELL) and lateral line fibers (LL) that extend to innervate migration populations of LL and some ELL. The ear is unique to vertebrates, giving rise to the VIII ganglia that innervate more ventral nuclei compared to LL and ELL projections to depend on *Atoh1* for hair cells, *Neurog1* and *Neurod1* for neurons. Note, the fly uses atonal for eyes and ears that is known as lin-23 in C. elegans. CP, choroid plexus; DRG, dorsal root ganglia; WGD, whole genome duplication. Modified after (Elliott et al., 2021, 2022; Fritzsch, 2021; Holland, 2020; Holland and Daza, 2018; Stolfi, et al., 2015; Tang, et al., 2013; Zhao, et al., 2020).

**Fig. 2:**
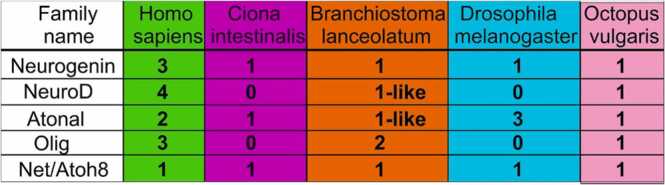
**.** basic Helix-Loop-Helic (bHLH) genes are found in all animals and plants and are shown here for only a small set of bHLH genes and species. Note that the total of bHLH genes of 13 (*Hs*), 3 (*Ci*), 6 (*Bl*), 5 (*Dm*) and 5 (*Ov*) are documented in various gene analysis. *Atoh*-like and *NeuroD*-like are incompletely understood in the lancelet. Note that the largest number of bHLH genes are known among vertebrates (Hs) which is reduced to just 3 bHLH genes in the ascidians (Ci). Modified after (Chen et al., 2011, Deryckere et al., 2021, Holland et al., 2008, Simionato et al., 2007).

In this review we contrast the sensory cell types and genetic control of cell diversity in the evolution of chordates. We propose a parallel duplication of certain basic Helix-Loop-Helix genes (bHLH) that are expressed near the brainstem and spinal cord (Fig. 1, Fig. 2) as well as the eyes (Fritzsch et al., 2015, Murre, 2019, Simionato et al., 2007). A single *atonal/lin-32/Atoh* bHLH gene is known in Protostomia (fly*, D. melanogaster;* nematode*, C elegans*) and Deuterostomia but is unclear in the lancelet (Holland, 2020, Holland et al., 2008, Simionato et al., 2007, Zhao et al., 2020). Emergence of the genes *Atoh* in sensory cells and *Neurog* in spinal cord has been demonstrated for ascidians (Cao et al., 2019, Stolfi et al., 2015, Tang et al., 2013). Furthermore, a duplication of *Neurog1/2* and formation of a bHLH gene, *Neurod1*, is associated with the emergence of distinct sensory cells (under control of *Atoh1*) and neurons (under control of *Neurog1/2; Neurod1*) in vertebrates (Fritzsch, 2021). In addition, we highlight the numbers of *Neurod1* (4 in vertebrates), *Neurog1* (3 in vertebrates), *Atoh/atonal/lin-32* (2 in vertebrates,3 in flies) and *Olig’s* (3 in vertebrates, 2 in the lancelet; Fig. 2). The absence of *Neurod1* and *Olig* (*C. intestinalis; D. melanogaster*) stands in contrast to to *Octopus vulgaris*, which has at least one bHLH gene [Fig. 2; (Deryckere et al., 2021; Simionato, et al., 2007)].

We propose that the two rounds of whole-genome duplications [WGD; (Holland and Daza, 2018)] which occurred near the base of the vertebrate lineage (around 580–530 million years ago; (Holland and Holland, 2022)), initially allowed a divergence between photoreceptor and projection neurons (RGCs) and subsequently between pineal and lateral eye structures to evolve *Neurod1* and *Atoh7* (Fritzsch et al., 2010).

### 1.1 Amphioxus (Lancelet)

**Fig. 3:**
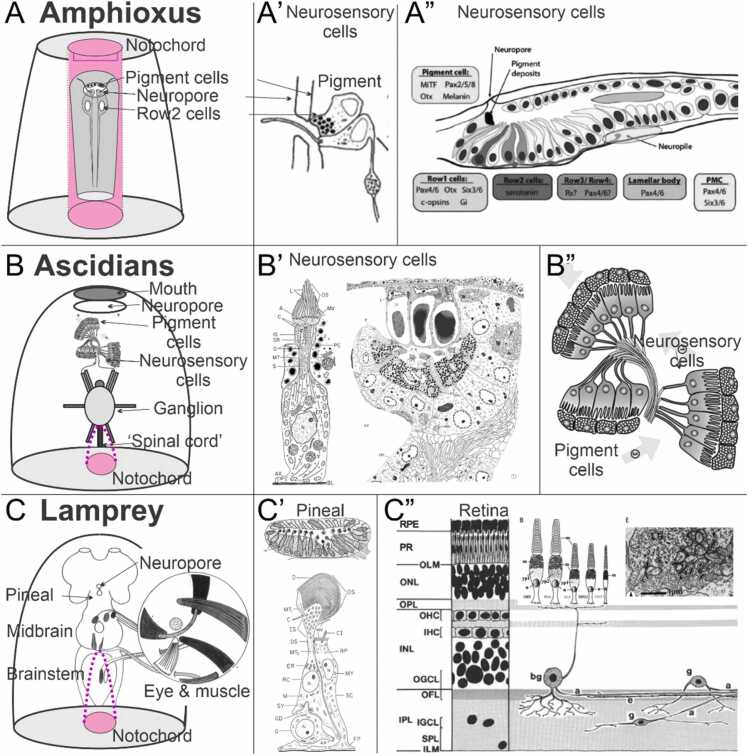
Comparing lancelet, ascidians, and lamprey eyes. (A) Lancelet has cilia without lamellae in a frontal eye (A’) that protrudes through the neuropore and has neurosensory cells that project rostrally (A’’). Note that the notochord (lilac) extends beyond the rostrum (section view) and contains pigment cells. (B) Ascidians have a short (or absent) notochord (lilac) and a neuropore which in adults will open through the mouth and possess single or complex cilia (B’) with an outer segment of lamellae. The outer segments are next to or separate from pigment cells (B’’) and project to the neural ganglion that may exhibit a ‘spinal cord’ in derived ascidians. (C) Lampreys have asymmetric pineal dorsal photoreceptor cells that connect with ganglion cells and form synapses. The eye is moved by three sets of extraocular muscles (inset). A transient neuropore opens anterior to the pineal (C’) and expands into a bilateral retina (C’’) containing rods and cones that interconnect with bipolar, horizontal, amacrine and ganglion cells, some of which extend to the outer plexiform layer. Modified after (Barnes, 1971, Braun and Stach, 2017, Braun and Stach, 2019, Collin et al., 2009, Eakin , 1973, Eakin and Kuda, 1970, Fritzsch and Collin, 1990, Fritzsch et al., 1990, Lacalli et al., 1994, Martin and Fritzsch, 2022, Suzuki and Grillner, 2018).

Among chordates, lancelets share with vertebrates a notochord, spinal cord, a neuropore and associated brain-like structures (Fig. 1, Fig. 2, Fig. 3). Lancelets did not experience the two whole genome duplications that underly the genetic makeup of vertebrates, and they possess fewer bHLH genes than humans do (Holland and Daza, 2018, Holland and Holland, 2022). Four different photoreceptive regions are present among lancelets: these are the frontal eye next to the neuropore, the lamellar body, the Joseph cells and the dorsal ocelli (Holland, 2020, Lacalli et al., 1994). The frontal eye has similarities to the retina, with specific gene expression domains having counterparts in the vertebrate retina (Pergner and Kozmik, 2017). To date it has not been confirmed that the ciliary cells of the frontal eye and lamellar body are light-responsive (Lamb, 2013). Moreover, the frontal eye receptors lack several other features of ciliary neurons. The Joseph cells and the dorsal ocelli cells have some features of protostome microvillar photoreceptors, and also express the enzymatic machinery of the transduction cascade, have established photoreceptor function with excitation maximum near 470 nm (del Pilar Gomez et al., 2009) and are connected with the frontal eye fibers (Lacalli, 2018). Immediately anterior to the neuropore are pigmented cells that express the opsin-related transcription factors *Pax2/5/8*, *Otx*, *Mitf* as well as melanin synthesis genes (Fig. 3; (Lamb et al., 2007)). Further from the pigmented cells are sensory cells positive for *Pax4/6* as well as *Otx*, *Six3/6*, and c-Opsins (Vopalensky et al., 2012). Each sensory cell has a ciliary process that extends toward and out of the neuropore. A central projection through an axon is forming which reaches a central target (Lacalli et al., 1994, Pergner and Kozmik, 2017) that may connect to the primary motor output (Lacalli, 2018, Schlosser, 2021).

Current data suggest the pigment regeneration chemistry in lancelets is homologous that in vertebrates (Albalat et al., 2012, Poliakov et al., 2012, Vopalensky et al., 2012). The chromophore covalently binds at position Lys 296 in all opsins (Lamb, 2013, Pasqualetto et al., 2020). Molecular fingerprinting of lancelets has identified a total of 21 opsin genes (Pergner and Kozmik, 2017) which produce highly variable opsin proteins: this diversity is comparable to that in vertebrates. The opsin gene family structure thus provides information on duplications and gene loss events in lancelet (Pantzartzi et al., 2018). Also in common with vertebrates, lancelets have genes for melanopsin that could be comparable to vertebrate melanopsin (Koyanagi et al., 2005, Lucas et al., 2020, Pantzartzi et al., 2017).

From the point of view of eye evolution, the central projections of vertebrate retina arise from ganglion cells that express the basic helix-loop-helix (bHLH) gene *Atoh7* (Brzezinski and Reh, 2015, Chan et al., 2020, Zhao and Peng, 2021). The *Atoh 7* gene is not present in the lancelet (Holland, 2020, Holland et al., 2008). A single *atonal*-like gene does resemble the *net* genes of *Drosophila* but is considered an *Orphan* bHLH gene rather than indicating an ancient origin of *Atoh 7* (Cao et al., 2019, Chen et al., 2011). Further, as shown in following sections, the ciliary sensory cells of vertebrates end in a synaptic ribbon which contacts secondary sensory cells and is also under control of *Atoh7* (Zhao and Peng, 2021)*.* Gene expression that resembles certain aspects of other chordates is known among lancelets (Holland, 2020). For example, *Foxg1, Emx, Otx,* and *Gbx* genes are common to lancelets and vertebrates (Cao et al., 2019, Glover et al., 2018). However, in vertebrates the transcription factor *Foxg1* has an early interaction with *Sox2* to control retina formation (Cao et al., 2019, Dvorakova et al., 2020) downstream of *Eya1/Six1* (Moody and LaMantia, 2015, Zhang et al., 2021).

In summary, beyond the early steps that produce a ‘frontal eye’ under control of certain primordial genes (*Pax2/5/6, Pax4/6, Six3/6, Otx, c-opsins*) in lancelets, the evolution of vertebrate eyes required major steps to transform a ciliated sensory neuron (lancelet) into the split arrangement of a ciliated sensory cell and a central projection neuron (RGCs in vertebrates). We suggest at least two or more additional genes evolved in vertebrates (*Atoh7, Neurod1*) to convert a single neurosensory, neuropil-projecting cell into a ciliary sensory cell connected with a neuropil-projecting ganglion cell.

### 1.2 Ascidians (Tunicates)

Over 3000 species of ascidians have been characterized (Braun et al., 2020, Winkley et al., 2020). Ascidians have a reduced genome of 70–170 million base pairs (Mbp) compared to lancelet (~ 520 million base pairs) and humans [~ 3 billion base pairs; (Fodor et al., 2021, Holland, 2020)]. Adults in two of five major ascidian groups possess a nerve cord, which is reduced compared to the lancelet [there are ~89 neurons in the near adult sea-squirt *Ciona* compared to ~20,000 neurons in the lancelet; (Cao et al., 2019, Holland, 2020, Ryan et al., 2018)]. We know that the telencephalon is absent and has no olfactory connections (Cao, et al., 2019). Ciliated sensory cells with adjacent pigment cells are present in nearly all ascidians, at least during early development (Braun et al., 2020, Konno et al., 2010, Kourakis et al., 2019, Winkley et al., 2020). The sensory cells have an outer segment with multiple lamellae (Fig. 3), and each sensory cell connects directly to the nearby neuronal ganglion (Braun and Stach, 2019, Konno et al., 2010, Lacalli and Holland, 1998, Lacalli et al., 1994, Ryan et al., 2018). Detailed analysis shows the origin of the neuropore in *Ciona* (Veeman and Reeves, 2015) and confirmed the independent connection of the neuropores with the opening of the gut (Veeman et al., 2010). The neuropore is fused with the mouth orifice, an arrangement that is prevented by the rostrally extended notochord of lancelets [Fig. 2; (Ota and Kuratani, 2006)].

In the free-floating ascidian salp *Thalia*, oocytes develop into a blastozooid stage within a few hours (Winkley et al., 2020). The oocyte has a single horseshoe-shaped set of pigmented cells that are adjacent to the receptor cells (Lacalli and Holland, 1998). However, in the blastozooid stage, the receptor cells form three distinct pigment-associated cups, each one pointing in a different direction (Braun and Stach, 2017). The cups have transparent transient lens cells (Barnes, 1971) and have three discrete groups of sensory cells of which only one is associated with melanin (Konno, et al., 2010). The simple brain of all larval ascidians is connected by multiple branches to these ‘eyes’ but the connections have not been analysed at the level of individual terminals (Braun and Stach, 2019, Ryan et al., 2018).

Among the two *Atoh*-like genes in ascidians, one is clearly related to *Atoh1* and develops with *Neurog* to innervate the sensory cells and reach out to the anterior motor ganglion (Cao et al., 2019, Fritzsch, 2021, Stolfi et al., 2015, Tang et al., 2013). Another gene, *Foxg1*, that is expressed in the olfactory system in vertebrates (Dvorakova, et al., 2020) is also expressed in the brain, which is housed in the anterior neural plate (*C. intestinalis;* Fig. 2*;* (Liu and Satou, 2019) and likely incorporated with the ectoderm to contribute to the vertebrate forebrain (Cao, et al., 2019). A gene expressed in the brain is currently equated with *Atoh8* (Negrón-Piñeiro et al., 2020) that is identified as an Orphan bHLH gene by others (Cao, et al., 2019). Moreover, *Atoh8* is split into three parts in chordates that make it difficult to align the genes (Chen, et al., 2011). *Atoh8* is expressed broadly in earliest mammals, is broadly expressed outside the brain in vertebrates (Rawnsley et al., 2013) and exerts only a limited effect on eye (Divvela et al., 2022) and ear development (Tang et al., 2021). Moreover, there is no expression of *Atoh*-like genes in the forebrain and *Orphan*-bHLH genes are present, suggesting no *Atoh* is present in *Ciona* [for example, *Pax2/5/8*. *Pou*, *Isl;* (Cao et al., 2019, Fritzsch and Piatigorsky, 2005)]. These data are consistent with the proposal that duplication of several bHLH genes through WGD enabled evolution of *Atoh1*/*Atoh7* in vertebrates (Fritzsch et al., 2010, Holland and Daza, 2018).

The sea squirt *Ciona* expresses an opsin-1 gene, which is closely related to the vertebrate visual opsins (Kusakabe et al., 2001, Pisani et al., 2020, Terakita, 2005). As intimated above for the lancelet, the ascidians are unable to transform all-*trans* to 11-*cis* retinal, relying instead on photoisomerization (Albalat et al., 2012, Poliakov et al., 2012). Interestingly, certain species do not bind RA while others use conventional RA signaling (Fujiwara and Cañestro, 2018). Further, the ascidian arrestin binds to opsin-based pigments, a function similar to the vertebrate non-visual arrestin, *β-arrestin* (Kawano-Yamashita et al., 2011, Nakagawa et al., 2002).

In summary, although certain similarities are clear with respect to opsins in the lancelet, ascidians and vertebrates, we lack a model of how to transform the simple frontal eye of lancelet to the tunicate eye (Lamb, 2013). The main difference is the lack of certain genes (*Pax4/6, Eya/Six, Mitf*) which are required for eye development at the level of vertebrates. On the other hand, studies of gene regulatory networks have revealed evolutionarily conserved gene circuits between ascidians and vertebrates (Cao et al., 2019, Liu and Satou, 2020, Ryan et al., 2018). Moreover, ascidians have neurosensory cells that are associated with pigment cells and likely depend on *Mitf* signals from the mesenchyme as is the case in lancelets and vertebrates. These similarities let us to propose a split into independent populations of sensory and projection cells in the ascidians must have happened at or near the base of the vertebrate lineage some 530 million years ago in line with the quadrupling of Hox clusters (Holland and Daza, 2018).

## 2 From lampreys and hagfish to jawed fishes: Retina development and evolution

As summarized above, from an evolutionary perspective, eye development starts with neuropore-associated sensory cells. We next consider eye and retina development as exemplified in the extant cyclostomes (lampreys and hagfish) that first appeared ~525 million years ago and have evolved into 35 lamprey and 79 hagfish species [OneZoom: Jawless vertebrates].

### 2.1 Lampreys

In lampreys, retina formation begins in the late embryos with a few receptors and retinal ganglion cells (RGC) that receive a prominent efferent input from the brain (Anadon et al., 1998, Barreiro‐Iglesias et al., 2017, De Miguel et al., 1989, Fain, 2019, Fritzsch, 1991, Fritzsch and Collin, 1990, Lamb, 2013, Pombal and Megías, 2019, Suzuki and Grillner, 2018, Villar-Cheda et al., 2006). Opsin-expressing photoreceptor cells are reported to be present early in larvae and to make contact with RGCs and bipolar cells (Suzuki and Grillner, 2018). If confirmed, this result means the developmental sequence of *RGC>horizontal>cone>amacrine>rod>bipolar* (Lamb, 2013 ), which is characteristic of jawed vertebrates (as described in detail below), is different to that in lampreys where there is simultaneous formation of *rods>RGC>bipolar* followed by horizontal and amacrine cells (Suzuki and Grillner, 2018). Lampreys (Fig. 3) express up to five different sensory cells (Collin et al., 2009, Warrington et al., 2021) with morphology resembling that of jawed vertebrates (Lamb, 2013, Lamb et al., 2007). One sensory cell type expresses rhodopsin and shows rod-like transduction (Lamb, 2013) yet also shows cone-like light saturation dynamics (Fain, 2019, Morshedian and Fain, 2017). In jawed vertebrates the retinal ganglion cells form a distinct layer but in lampreys the majority (~74%) of RGC somas are in the inner nuclear layer (INL) (Fritzsch and Collin, 1990, Jones et al., 2009) and, unlike the arrangement in jawed vertebrates, in lampreys the optic fibers run scleral to the inner plexiform layer (Fritzsch and Collin, 1990). Further, some ganglion cells make direct contact with photoreceptors: a situation otherwise unprecedented in gnathostomes (Fain, 2019, Jones et al., 2009). The different position of RGCs (IPL versus GCL) combined with branches that reach photoreceptors may indicate a unique and direct input of cones to RGCs in lampreys (Fritzsch and Collin, 1990, Jones et al., 2009, Suzuki and Grillner, 2018). On the other hand, recent electrophysiological data (Morshedian et al., 2021) shows the classical vertebrate split into On-type and Off-type light response polarity is present in lamprey retina.

Recent data confirmed earlier interaction in the cerebellum between *Atoh1* and *Neurod1* (Kersigo et al., 2021, Sugahara et al., 2021). Unfortunately, *Atoh7* is known to be present but has not been analysed in the lamprey (Smith et al., 2013) in which there is expression of *Neurod1* in the eye and the pineal is needed.

### 2.2 Hagfish

Available evidence suggests that unlike lamprey eye, the hagfish eye does not have a lens (Baden et al., 2020, Collin and Fritzsch, 1993, Dong and Allison, 2021, Gustafsson et al., 2008, Song and Kim, 2020), which is now clarified as playing a major role in eye development (Fain, 2020, Fuhrmann, 2010). If confirmed, the hagfish eye would be the only extant eye without a lens (Miyashita, 2020, Schwab, 2018). The ciliated sensory cells in hagfish require rhodopsin and are likely rod cells (Lamb, 2013): a suggestion consistent with recent evidence for true rods and cones in lampreys (Fain, 2019, Fain, 2020). The retinal ganglion cell disposition resembles that of lampreys (Fritzsch and Collin, 1990, Suzuki and Grillner, 2018), likely correlated with the absence of myelin (Fritzsch and Glover, 2007), and there may be a direct input from ciliated sensory cells to RGCs via ribbons (Holmberg, 1971, Holmberg, 1977, Locket and Jørgensen, 1998). Its similarity with the brainstem expression in the hagfish (Higuchi et al., 2019) makes it likely to be positive for *Atoh7* and *Neurod1* in the eye expression.

### 2.3 Gnathostomes

The sequence of development of different retinal cell types in gnathostomes (jawed vertebrates) has been established following the initial discovery of proliferation of a column of retinal cells (Cepko et al., 1996). The earliest stage is the formation of RGCs: this process depends on the transcription factor *Atoh7* in all vertebrates (Baker and Brown, 2018, Wu et al., 2021, Zhao and Peng, 2021). Downstream of *Atoh7* are *Brn3b*/*Pou4f2* and *Isl1* and the *distalless homolog* (*Dlx2*) which is not regulated by *Atoh7* (Brzezinski and Reh, 2015, Cao et al., 2019). The next stage is generation of cone and amacrine cells, followed by horizontal cells. Rod and cone cells depend on the transcription factor *Neurod1* (Dennis et al., 2019, Fritzsch et al., 2010, Kersigo et al., 2021, Neitz et al., 2021) and interactions with other genes to allow their differentiation as distinct sensory cells of cone and rods such as *Crc* (Brzezinski and Reh, 2015, Wu et al., 2021). The best-studied species are zebrafish, *Xenopus*, chicken and (predominantly) mouse (Baker and Brown, 2018, Hutcheson et al., 2005).

A detailed description of retinal development is provided by Fuhrmann (2010), highlighting multiple steps involved in retina expansion and interaction with the lens (Fig. 3). The eye field in the neural plate first splits into two parts which each interact with the lens placode to form a retina. As the retina expands it induces development of retinal pigment epithelium (RPE). The next steps are driven by mesenchymal induction of the eye field transcription factors *Mitf, Pax6, Rax, Otx2, Six3* and *Lhx2* (Fuhrmann, 2010)*.* In addition, the fibroblast growth factor *Fgf* is secreted from the lens ectoderm (Fuhrmann, 2010) and upregulates *pERK* signal transduction genes as well as transcriptional regulators *Vsx2* and *Sox2.* Expression of *Sox2* drives the neuronal part of the retina development, requiring *Vsx2*-mediated suppression of *Mitf* (Fuhrmann, 2010).

Once the lens begins forming, three distinct areas (RPE, neural retina, optic stalk) require additional interactions under wingless / integrated (*Wnt*) signal transduction pathways to retain RPE under *Mitf, BMP, RA* and *β-catenin* control (Fig. 2), and interaction with *Shh* to define the optic stalk (Buono and Martinez‐Morales, 2020, Fuhrmann, 2010). These complex interactions have been detailed in *Xenopus* with emphasis on the role of *noggin* as an antagonist for *BMPs* involving *tbx3, Pax6, Pax2, Vsx2, Shh* and RA (Sasagawa et al., 2002). Comparable information exists for early ear development in the zebrafish for which interesting effects of the same controlling genes can be studied in context of visual development, anophthalmia, retinitis pigmentosa, cyclopia and coloboma (Cavodeassi et al., 2005, Richardson et al., 2017). Unfortunately, much work on cyclostomes is still required to fill in the gaps in our understanding.

In summary, a set of intrinsic and extracellular factors control eye organogenesis but there are gaps in knowledge of these interactions, in particular the control of evagination of the retina and the specific roles of RA and other genes (Fuhrmann, 2010, Lamb, 2013, Manns and Fritzsch, 1991).

## 3 Pineal sensory system

A parallel sensory system to the retina is the pineal and parapineal of lampreys and gnathostomes; these structures are also known as parietal or pineal eyes (de Vlaming and Olcese, 2020, Koyanagi et al., 2004, Meléndez‐Ferro et al., 2002, Pu and Dowling, 1981, Reiter, 1980, Roberts, 1978, Simonneaux and Ribelayga, 2003, Vigh-Teichmann et al., 1983). Pineal eyes are absent in hagfish (Lamb, 2013). A different pattern of connections is present in mammals compared to non-mammalian vertebrates (Reiter, 1980, Simonneaux and Ribelayga, 2003). Melatonin is a hormone that is secreted by the pineal and plays a role in the biorhythms and reproductive activity in mammals (Kim et al., 2022).

The pineal forms adjacent to the neuropore and represents a dorsal expansion of the diencephalon. In the pineal, ciliated sensory cells connect to ganglion cells with ribbon synapses (Lamb, 2013, Pu and Dowling, 1981). The pineal receptors have rod-like function and use rhodopsin (Lamb, 2013). The presence of two cell types (ciliated sensory and ganglion cells) sets the pineal apart from the direct neurosensory output in lancelet and ascidians (neurosensory cells), and suggests that pineal eyes evolved together with or following the evolution of lateral eyes with RGCs (Fain, 2019, Jones et al., 2009). The left and right pineal and parapineal in lampreys and several gnathostomes have different sizes (de Vlaming and Olcese, 2020, Pu and Dowling, 1981, Smith et al., 2018). The pineal is connected with the right whereas the parapineal is connected to the left, forming an asymmetric habenula (Meléndez‐Ferro et al., 2002, Puzdrowski and Northcutt, 1989). The presence of three distinct receptor clusters in tunicates (Braun and Stach, 2019) could be the base of the pineal that has been equated with ascidians split into three eye clusters (Horie et al., 2008, Negrón-Piñeiro et al., 2020, Schlosser, 2021).

Several genes are associated with the mammalian pineal including *Lhx9, Pax6* and *Otx2* (Yamazaki et al., 2015, Yang et al., 2019). The expression of *Neurod1* is documented (Coon et al., 2019, Ge et al., 2020, Munoz et al., 2007) but the pineal appears histologically normal after *Neurod1* deletion (Ochocinska et al., 2012). A limited expression of *Atoh7* in *Xenopus* pineal has been shown (Hutcheson, et al., 2005); resolving these differences will require a detailed analysis in some of the fully developed vertebrates and mammals (which show a different organization of the pineal).

## 4 Neuropore formation is unique among chordates

**Fig. 4:**
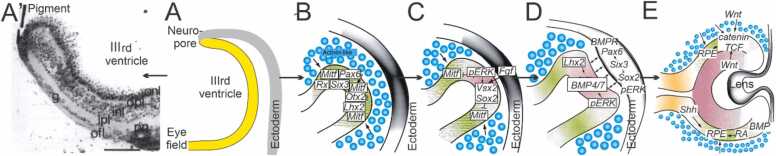
Molecular control of retina development. Retina formation starts ventral to the neuropore, adjacent to the IIIrd ventricle and shows expression of the eye field (A). Right panels display sequential upregulation of transcription factors *Mitf* and *Rax* to interact with *Pax6*, *Six1*, *Otx2* and *Lhx2* (B) followed by *Fgf* expression collaborating with *Sox2* to suppress *Mitf* (C). Several genes interact to upregulate the lens placode during eye formation (D). *Shh* defines the optic stalk, *Mitf* develops pigments whereas *Sox2* and *Pax6* induce neuronal precursors in the retina (E). (A’) shows the effect of high doses of *RA* causing the evagination to be suppressed but a normal retina develops adjacent to the pigment. Modified after (Buono and Martinez‐Morales, 2020, Fuhrmann, 2010, Manns and Fritzsch, 1991).

**Fig. 5:**
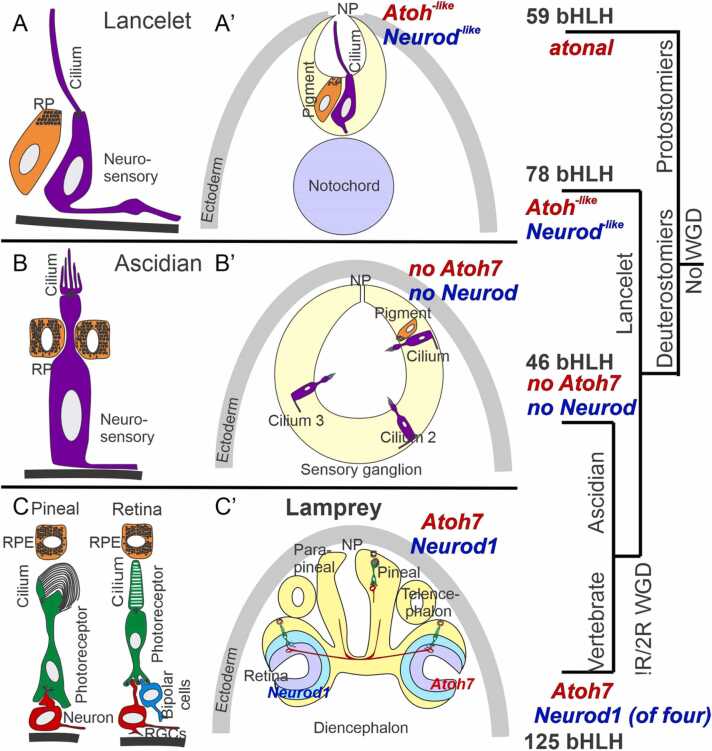
Comparison of sensory cilia formations in chordates. Lancelet has a simple cilium (A) that is located adjacent to pigment cells (A’). The central lumen of the terminal sac extends cilia into the neuropore (NP). The ‘sensory ganglion’ comprises one cilium associated with pigment and two ciliary sensory cells that are not associated with pigments. Ascidians have cilium branches (B), tightly associated pigment cells, and projections to the neuropore comprising three groups of pigment cells in certain ascidians (B’). Lampreys have two photoreceptive organs: the pineal/parapineal and the retina (C). Photoreceptor cells in both organs terminate in ribbons (C). Brain projections arise either directly (pineal) or from ganglion cells (RGCs) in a retina that also has bipolar cells, horizontal and amacrine cells to process the visual input and transmit it to the brain. Note that vertebrates have distinct bHLH genes (*Atoh7*, *Neurod1*, C’) that are not found in the lancelet (A’) nor the ascidians (B’). atonal is needed for eye development in flies and possibly evolved into 4 distinct genes in vertebrates. Modified after (Cao et al., 2019, Elliott et al., 2021, Fritzsch et al., 2015, Holland, 2020, Martin and Fritzsch, 2022, Suzuki and Grillner, 2018).

A common feature in chordates is the formation of the neuropore that transiently connects with the rostral neural tube and the external skin (Fig. 3, Fig. 4, Fig. 5). Formation of the neural tube starts in the hindbrain and fusion progresses anteriorly, leaving the neuropore at the last step of closure the dorsal part of the brain in gnthostomes (Maniou et al., 2021). Likewise, he ‘frontal eye’ in lancelets exhibits a few sensory cells which reach out of the neuropore (Holland, 2020, Lacalli et al., 1994). A detailed description in ascidians shows the formation of a neuropore that opens to the mouth directly (Veeman and Reeves, 2015, Veeman et al., 2010). In lampreys the neuropore starts as a keel that forms into the neural tube in later development (Richardson et al., 2010, Sauka-Spengler and Bronner-Fraser, 2008). A neuropore has been described for hagfish but not much further information is available due to limited knowledge of hagfish development (Higuchi et al., 2019, Ota and Kuratani, 2006, Wicht, 1996). Overall, this early step is comparable to the keel of zebrafish neuropore formation and similar factors may influence closure of the neuropore (Shinotsuka et al., 2018) in amniotes (Copp and Greene, 2010, Maniou et al., 2021, Müller and O'Rahilly, 1987, Rolo et al., 2019).

The genes *Opb* (open brain) and *Zic2* interact with *Shh* to regulate closure of the neural tube and reduced eye development is one consequence of failure of neural tube closure (Eggenschwiler et al., 2001, Gunther et al., 1994, Guo et al., 2006, Rolo et al., 2019). In part, the absence of *Msx1* and *Wnt3a* underlies the effects seen in *Opb* and *Zic2* null mice, in which the brain remains open without any closure (Copp and Greene, 2010). Downstream to *Opb/Zic2* are *Fgfs, Wnts*, Retinoic acid (*RA*), *Nodal* and *BMPs* that provide the signals that will, eventually, close the neuropore (Cao et al., 2019, Wilson and Houart, 2004) and are involved with *Shh* in mutants (Eggenschwiler et al., 2001). Triazole interferes with RA signaling and produces numerous defects in retinal and anterior neuropore formation as well as otic defects (Shinotsuka, et al., 2018). *Wnt/Frizzled* signaling involves a set of the planar cell-polarity genes (*Vang, Scrb1, Crash, Disheveled*) that cause the craniorachisis in mutant mice, implying these signal pathways in closure of the neuropore (Rolo, et al., 2019). In summary, the proximity of the neuropore to the pineal/parapineal in lancelet, ascidians, mice and man can affect the pineal and retina in extreme cases after loss of *Opb* to connect with craniorachisis.

## 5 A plausible sequence for retina development and evolution

A new hypothesis of retina evolution is proposed (Fig. 5). Building on previous work describing the formation of two distinct cell types through whole genome duplication (Fritzsch and Elliott, 2017, Holland and Daza, 2018), we can envision the same process permitting eye development through retina multiplication (Fig. 5). The vertebrate genome duplicated twice prior to divergence of the lineage that became vertebrates from other chordates (Fig. 2): this whole-genome duplication permitted the splitting of sensory cells from brain-projecting neurons in lampreys as well as gnathostomes (Holland and Daza, 2018, Smith et al., 2013). The alternative hypothesis that multiple cell types existed in ancestral organisms and were then lost in lancelets and tunicates cannot be ruled out, but the weight of evidence summarized in the foregoing sections is in favor of the splitting hypothesis.

We now propose that the increased gene pool also allowed divergence of more complex visual structures, that is, lateral eyes and pineal that develop next to the neuropore. Neurosensory cells have dual function of light transduction and central projection, represented by cells that have a ciliary process with its own axon in the lancelet and ascidians. These cell types form three distinct clusters in the blastocyst life stage of the tunicate sea squirt *Thalia*. We propose two different sized clusters in *Thalia* and *Ciona* evolved into pineal/parapineal and the third, largest, cluster evolved into the lateral eyes of vertebrates. By analogy with a split into distinct cell types (sensory cells and ganglion cells) from a single cell type (neurosensory cells) in lancelet and ascidians (Fritzsch, 2021, Fritzsch and Elliott, 2017, Holland and Daza, 2018), we suggest a split into distinct retina-like structures that is shown by the presence in lampreys of the pineal, parapineal, and bilateral retinas proper. An intermediate stage of this process may be represented by the direct neuropil projections of sensory cells in certain lampreys and hagfish. The divergence in lamprey of two kinds of ciliated sensory cells (under control of *Neurod1*) and a set of neurons (under control of *Atoh7*) is proposed to parallel that in gnathostomes, which subsequently have developed many more basic neuron types (Lamb, 2013). Further work is needed to detail the three vertebrate *Atoh (Atoh1, Atoh7, Atoh8*) and four *Neurod (Neurod1, Neurod2, Neurod4, Neurod6*) genes that will allow to establish the bHLH genes in chordates.

## 6 Summary and conclusion

Central projection of visual information in vertebrates has its evolutionary origin in a single neurosensory, central projecting, cell type. This single cell type split into a sensory ciliated cell (*Neurod1*) connecting via ribbons to the *Atoh7*-regulated cell families that send the visual information to the brain. The three different branches of vertebrate eyes (retina, pineal, parapineal) could have originated in the three retinal outputs present in ascidians that all develop near the neuropore.

## Author Contributions

BF and PM wrote the manuscript. All authors read and approved the final manuscript.

## Funding

BF was supported by 10.13039/100000002NIH/10.13039/100000049NIA (R01 AG060504).

## Declaration of Competing Interest

The authors declare that the research was conducted in the absence of any commercial or financial relationships that could be construed as a potential conflict of interest.
